# Biodegradable Cardiac Occluder with Surface Modification by Gelatin–Peptide Conjugate to Promote Endogenous Tissue Regeneration

**DOI:** 10.1002/advs.202305967

**Published:** 2023-11-20

**Authors:** Pengxu Kong, Xiang Liu, Zefu Li, Jingrong Wang, Rui Gao, Shuyi Feng, Hang Li, Fengwen Zhang, Zujian Feng, Pingsheng Huang, Shouzheng Wang, Donglin Zhuang, Wenbin Ouyang, Weiwei Wang, Xiangbin Pan

**Affiliations:** ^1^ Department of Structural Heart Disease National Center for Cardiovascular Disease China & State Key Laboratory of Cardiovascular Disease Fuwai Hospital Chinese Academy of Medical Sciences & Peking Union Medical College National Health Commission Key Laboratory of Cardiovascular Regeneration Medicine National Clinical Research Center for Cardiovascular Diseases Beijing 100037 China; ^2^ Tianjin Key Laboratory of Biomaterial Research Institute of Biomedical Engineering Chinese Academy of Medical Sciences and Peking Union Medical College Tianjin 300192 China; ^3^ Key Laboratory of Innovative Cardiovascular Devices Chinese Academy of Medical Sciences Beijing 100037 China

**Keywords:** biodegradable polymers, cell–material interface, congenital heart disease, occluder, tissue regeneration

## Abstract

Transcatheter intervention has been the preferred treatment for congenital structural heart diseases by implanting occluders into the heart defect site through minimally invasive access. Biodegradable polymers provide a promising alternative for cardiovascular implants by conferring therapeutic function and eliminating long‐term complications, but inducing in situ cardiac tissue regeneration remains a substantial clinical challenge. PGAG (polydioxanone/poly (l‐lactic acid)–gelatin–A5G81) occluders are prepared by covalently conjugating biomolecules composed of gelatin and layer adhesive protein‐derived peptides (A5G81) to the surface of polydioxanone and poly (l‐lactic acid) fibers. The polymer microfiber–biomacromolecule–peptide frame with biophysical and biochemical cues could orchestrate the biomaterial–host cell interactions, by recruiting endogenous endothelial cells, promoting their adhesion and proliferation, and polarizing immune cells into anti‐inflammatory phenotypes and augmenting the release of reparative cytokines. In a porcine atrial septal defect (ASD) model, PGAG occluders promote in situ tissue regeneration by accelerating surface endothelialization and regulating immune response, which mitigate inflammation and fibrosis formation, and facilitate the fusion of occluder with surrounding heart tissue. Collectively, this work highlights the modulation of cell–biomaterial interactions for tissue regeneration in cardiac defect models, ensuring endothelialization and extracellular matrix remodeling on polymeric scaffolds. Bioinspired cell–material interface offers a highly efficient and generalized approach for constructing bioactive coatings on medical devices.

## Graphical abstract

In this study, the surface of the biodegradable cardiac occluder is modified by covalently conjugating biomolecules composed of gelatin and layer adhesive protein‐derived peptides, which promotes in situ tissue regeneration by accelerating surface endothelialization and regulating immune response in a porcine atrial septal defect model.

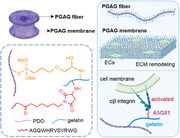

## 1 Introduction

Congenital heart defects (CHDs) refer to the general structural abnormality of the heart or thoracic great vessels at birth, which is the commonest congenital disease with an incidence of 4–50 per 1000 live births.^[^ ^1^ ^]^ Over the past decades, the therapeutic paradigm for CHDs has shifted from open‐chest surgery to minimally invasive transcatheter procedure.^[^ ^2^ ^]^ Currently, four main types of CHDs can be cured by transcatheter closure, including atrial septal defect (ASD), patent foramen ovale (PFO), patent ductus arteriosus (PDA), and ventricular septal defect (VSD).^[^ ^1^, ^2^, ^3^ ^]^ Biodegradable biomaterials aimed at inducing native tissue regeneration offer a promising alternative to cardiovascular implants compared to the formation of severe complications caused by metal‐based implant occlusion, such as cardiac perforation, erosion, rupture, arrhythmia and thrombosis.^[^ ^4^ ^]^ Recently, biodegradable occluders made of polydioxanone (PDO) and poly (l‐lactic acid) (PLLA) have been testified in clinical trials, suggesting the feasibility and safety of polymer‐based implants to occlude the defect.^[^ ^5^ ^]^

Most degradable polymer materials used in occluders including polylactic acid and PDO, have a relatively fast degradation rate in comparison with nitinol, and the degradation of biomaterials leads to a loss of mechanical strength, which therefore requires rapid tissue regeneration to withstand load transfer. However, such polymers have high crystallinity and high hydrophobicity,^[^ ^6^ ^]^ which are not advantageous to cell adhesion and migration. Degradable polymers are generally relatively plastic,^[^ ^7^ ^]^ and if the biophysical properties of the biomaterial do not match those of the local tissue, it can lead to loss of regenerated tissue and loosening of the polymeric implants. Occasional complications related to the material design have been reported in previous studies. Residual shunts developed in 3 of the 5 patients with degradable occluder implantation, which was increased during the 3 year follow‐up.^[^ ^8^ ^]^ Meanwhile, device malformation with disc expansion was observed in one case, which could be attributed to incomplete endothelialization after degradation initiation.^[^ ^8^ ^]^ Sievert et al. reported that moderate or large shunt occurred in 3 of 15 patients with poly(lactic‐co‐glycolic) acid (PLGA) occluders after implantation for 2 years.^[^ ^9^ ^]^ Therefore, the degradation rate of biomaterials should match the rate of in situ tissue regeneration for optimal nascent tissue growth and material–tissue integration, yet the balance between these two relationships remains clinically elusive based on previous cases. Additionally, the inflammatory response raised by polymer is still a risk for tissue erosion and rhythm disturbance. Our pre‐clinical studies also suggested that PDO occluder still induced transient arrhythmia and the frequencies decreased with device degradation and the relief of inflammatory response in a canine VSD model.^[^ ^5d^ ^]^ Moreover, 3 of 54 patients implanted with PDO occluder developed persistent right bundle branch block in a randomized controlled study during 2 year follow‐up.^[^ ^5b^ ^]^ Surface coating on degradable implant was used to improve material biocompatibility, such as hydrogel coatings.^[^ ^10^ ^]^ However, hydrogel coatings are not suitable for percutaneous catheter interventional delivery of occluders implanted in cardiac defects, due to the uncontrolled coating thickness that leads to the loss of hydrogel body structure during interventional therapy and the decrease of elasticity of the occluder itself.

Biomaterials interact with cells through their biophysical and biochemical properties, which can regulate the local tissue microenvironment by recruiting endogenous cells including immune cells, and endothelial cells, to guide the process of in situ tissue regeneration.^[^ ^11^ ^]^ As for the surface properties of biomaterials, the improvement of hydrophilicity ensures the promotion of albumin adsorption, leading to the local macrophages to produce anti‐inflammatory cytokines, which promotes the regenerative repair of damaged tissues.^[^ ^12^ ^]^ Optionally, biocompatible natural macromolecules are modified on the surface of scaffold materials to regulate cell‐biomaterial interactions and enhance cell spreading.^[^ ^13^ ^]^ Engineered biomaterials that guide the rapid recruitment, adhesion, migration, and infiltration of endogenous cells through biochemical pathways are critical for promoting in situ tissue regeneration.^[^ ^14^ ^]^ Biomaterials decorated with cell adhesion proteins presenting in the native extracellular matrix (ECM) enable biochemical signaling with cells through the recognition of these ECM proteins by cell surface receptors, including integrins.^[^ ^15^ ^]^ Therefore, we hypothesize the designed bioactive polymers to be decorated on the surface of implant materials, serving as biophysical and biochemical cues, would dictate cell adhesion and proliferation, and ECM remodeling on the polymer scaffolds, to induce endogenous tissue regeneration.

**Figure 1:**
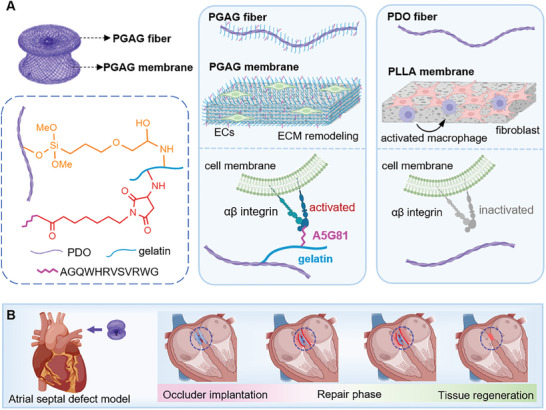
Schematic illustration for the preparation and application of PGAG occluder. A) PGAG occluder is composed of a PGAG frame and a PGAG flow‐blocking membrane. The gelatin‐peptide structure interacts with integrin receptor, promotes endothelial cell (EC) adhesion and proliferation, and mitigates pro‐inflammatory macrophage activation and fibrosis. B) Follow‐up of a porcine ASD model demonstrates cardiac repair induced by implantation of a PGAG occluder.

Herein, to address the unmet need of matching the degradation rate of implants to the rate of endogenous tissue regeneration, a bioactive molecular dressing was decorated on the surface of degradable PDO occluders, which consist of a PDO frame and PLLA barrier film. The PDO occluder decorated with bioactive molecules is identified as PGAG occluder (**Figure** **1**A). The polymer consists of gelatin covalently bonded with the laminin‐derived A5G81 peptide via Michael addition reaction. This peptide sequence (AGQWHRVSVRWG) is the cell adhesion domain in laminin that specifically interacts with integrins α3β1 and α6β1.^[^ ^16^ ^]^ Gelatin provides a biocompatible matrix for cell spreading, which is conducive to cell proliferation and growth,^[^ ^17^ ^]^ while A5G81 peptide is introduced to enhance the structural accuracy of the interaction with cells. The bioactive structure results in sustained activation of cell–material interface by providing more integrin adhesion sites. Moreover, the active dressing induced an anti‐inflammatory phenotypic differentiation of immune cells, inhibited inflammatory responses, and produced lower foreign body responses in vivo. The treatment with newly prepared occluder, without drug or cytokine, significantly induced heart tissue regeneration in ASD models (Figure 1B). This bioactive modification on the surface of biodegradable materials exhibited optimized cell‐biomaterial interactions while retaining the mechanical properties and dimensions of the implant, providing a broad design platform for next‐generation biodegradable medical devices that powerfully induce endogenous tissue regeneration and material–tissue fusion.

## 2 Results

### 2.1 Preparation and Characterization of PGAG

PGAG occluder is manufactured through the surface modification of PDO occluder decorated with bioactive polymers, including the flow blocking membrane, and the frame. The synthesis of bioactive polymers involved the coupling of gelatin with Maleimide‐terminated A5G81 (Mal‐A5G81) peptide covalently via amino‐maleimide click chemistry reaction (Figure S1, Supporting Information). A5G81 (AGQWHRVSVRWG) is a 12‐amino acid sequence screened from the soluble peptides from the laminin α5 chain G domain sequences, which is a cell adhesion domain of laminin.^[^ ^16^, ^18^ ^]^ The molecular weights of gelatin‐A5G81 and gelatin were determined by gel permeation chromatography (GPC). The Mp (molecular weight of the highest peak) of gelatin‐A5G81 and gelatin were 9913 and 9106 g mol^−1^, respectively, indicating that 0.52 mol of A5G81 peptide were bonded to 1 mol of gelatin (Figure S2, Supporting Information). To further confirm the amino‐maleimide click reaction, the amino acid concentration of gelatin and gelatin‐A5G81 was determined by ninhydrin reaction. The concentration of amino acid in 1 mg mL^−1^ gelatin was 3.03 µg mL^−1^, while the concentration of amino acid in gelatin‐A5G81 was 1.77 µg mL^−1^, which suggested that the concentration of amino groups in gelatin‐A5G81 decreased after Mal‐A5G81 was bonded. Furthermore, the ultraviolet–visible (UV–vis) spectrum showed that the Mal‐A5G81 possessed an absorbance peak at 300 nm, which corresponds to the absorption peak of the maleimide group,^[^ ^19^ ^]^ while gelatin‐A5G81 did not, which indicated that the maleimide group was fully reacted (Figure S3, Supporting Information).

**Figure 2:**
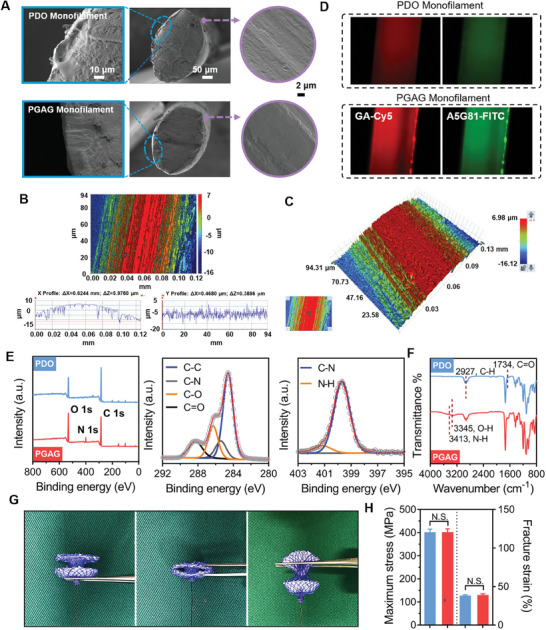
Characterization of surface structure and mechanical properties of PGAG. A) Cross‐sectional and lateral SEM micrographs of PGAG monofilaments. B,C) Surface optical profile images of PGAG monofilaments with longitudinal grooves. D) Fluorescent images of GA and A5GB1 of PGAG labeled with Cy5 and FITC, respectively. E) XPS survey scan, C 1s and N 1s spectra of PGAG. F) FTIR spectra of PGAG. G) Morphology of the PGAG occluder under compression and tension. H) The maximum stress, fracture strain for PGAG and PDO (*n* = 3). Data are presented as mean ± SD. *p*‐values are calculated using unpaired t‐test. N.S. means no significant.

PDO occluder was processed through oxygen surface plasma treatment and modified with a silane coupling agent containing an epoxy group. The silane pre‐treatment has been an innovative modification of cardiovascular implants, which degrade into single molecule of Si(OH)_4_ and would be removed by the urinary system without adverse reactions.^[^ ^20^ ^]^ Subsequently, gelatin‐A5G81 polymer was covalently grafted onto the occluder material surface through the ring‐opening reaction of amino and epoxy groups. Therefore, PGAG was synthesized by PDO grafting with Gelatin‐A5G81 (Figure 1A). The surface morphology of PGAG monofilaments was observed. Scanning electron microscopy (SEM) images of PGAG monofilaments showed a uniform diameter size and relatively obvious longitudinal grooves (**Figure** **2**A). Surface optical profile analysis revealed the existence of longitudinal grooves with a depth of 5–15 µm and groove width of 2–8 µm, uniformly distributed on the surface of PGAG frame (Figure 2B,C).

Furthermore, the presence of bioactive polymers on the surface of the PDO monofilament was characterized by various analytical methods. Bioactive polymers were visualized on the monofilament surface by molecularly labeling gelatin and A5G81 with Cy5 and FITC fluorescent dye, respectively, and further analyzed by fluorescence microscopy (Figure 2D). Results showed that strong red and green fluorescence on the surface of PGAG monofilaments were observed, clearly reflecting the continuous and stable bonding of bioactive molecules on the surface of the monofilaments. In addition, the surface chemical element of a representative PGAG characterized by X‐ray photoelectron spectroscopy (XPS) indicated the appearance of N peaks due to the bonding of gelatin and peptide, with the strongest characteristic peak attributed to nitrogen in N─C bonds, and the weaker peak corresponding to nitrogen in N─H bonds (Figure 2E). The GPC traces showed that the Mp of PGAG and PDO were 119460 and 108130 g mol^−1^, respectively (Figure S4, Supporting Information), indicating that 1.14 mol of gelatin‐A5G81 were bonded to 1 mol of PDO molecule and therefore, 0.59 mol of A5G81 peptide were bonded to 1 mol of PDO molecule. Fourier transform infrared spectroscopy (FTIR) spectrum also showed the characteristic stretching vibration absorption peaks at 3345 cm^−1^ and 3413 cm^−1^ assigned to O─H and N─H bonds in gelatin and A5G81, respectively (Figure 2F). These data suggested the successful preparation of peptide‐gelatin conjugated PDO occluder.

The mechanical properties of cardiovascular occluders grafted with bioactive polymers were further investigated. As shown in Figure 2G, the prepared PGAG occluder had good elasticity and deformability, which facilitated the transcatheter delivery of the occluder. PGAG monofilaments showed the same tensile strength and elongation at break as PDO monofilaments without significant differences (Figure 2H), indicating a favorable adaptability and reliability of this continuous modification process for fabricating degradable implants. Modification of the surface of implants with bioactive polymers affects their surface physical properties. PGAG material demonstrated a water contact angle of 20.7° (Figure S5, Supporting Information), which is significantly lower than that of native PDO material (87.1°). The improved hydrophilicity may facilitate the binding of extracellular matrix molecules to the material surface and enhance cell adhesion. Furthermore, the modification of gelatin and peptides slightly increased the glass transition temperature of PDO from −11.3 °C to −10.3 °C (Figure S6, Supporting Information), which may be attributed to the facile hydrogen bonding interactions between gelatin and polyester molecules, thus confining the mobility and molecular state of the polyester chains. Wide‐angle X‐ray diffraction (XRD) analysis demonstrated that the modification of the bioactive polymer did not affect the crystalline nature of the polyester (Figure S7, Supporting Information). These results indicate surface modification of PDO fibers has no effect on the mechanical property of occluder, while significantly promotes the hydrophilicity.

### 2.2 Promotion of Adhesion and Proliferation of Endothelial Cells on PGAG

**Figure 3:**
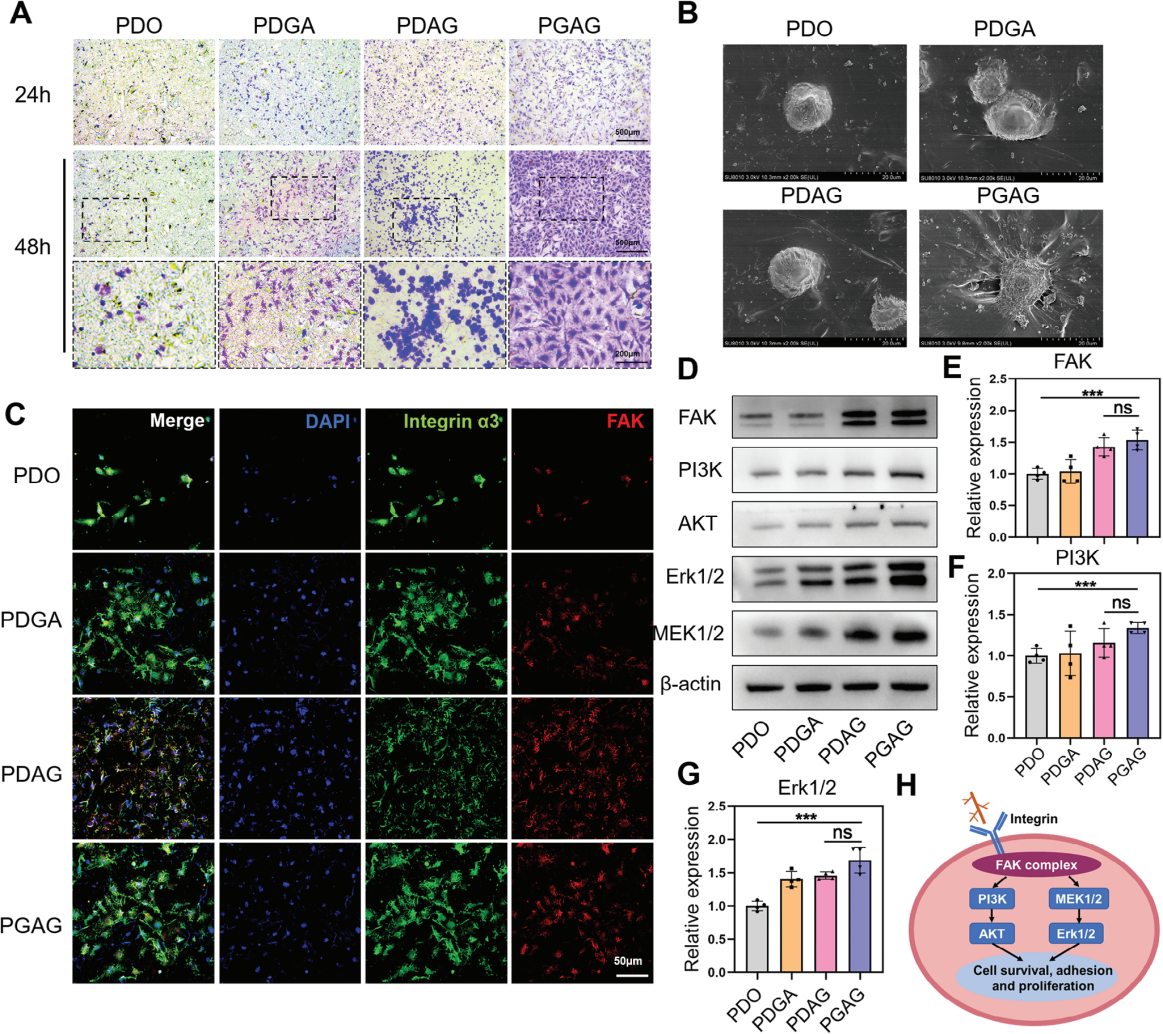
PGAG promoted survival, adhesion, and proliferation of endothelial cells. A) HUVECs cocultured on PDO, PDGA, PDAG and PGAG membranes for 24 and 48 h. B) Representative SEM images of HUVECs adhering on membranes. C) Representative immunofluorescence images of HUVECs cocultured on PDO, PDGA, PDAG and PGAG membranes (blue: nuclear; green: integrin α3; red: FAK). D) Western blot analysis of FAK, PI3K, AKT, Erk1/2, MEK1/2 protein expression by treated HUVECs. E) Quantitative expression of FAK, F) PI3K, and G) Erk1/2 (*n* = 4 for each test). H) Schematic diagram of PGAG promoting survival, adhesion, and proliferation of endothelial cells. Data are presented as mean ± SD. *p*‐values are calculated using one‐way ANOVA with Bonferroni correction. ns = no significance, *p < 0.05, **p < 0.01, and ***p < 0.001.

The biological cues of endothelial cells on biomaterials, including activation of survival, adhesion, migration, and proliferation, are closely associated with the process of endothelialization.^[^ ^21^ ^]^ PDO is highly hydrophobic, which is not advantageous to cell adhesion and infiltration. The ECM‐mimic structure of gelatin interface was expected to provide 3D porous microenvironment for cell adhesion and proliferation.^[^ ^17^, ^22^ ^]^ Moreover, the laminin‐derived A5G81 peptide is a potent tethered cell adhesion‐, proliferation‐, and endothelium‐inducing ligand that interacts specifically with the integrin receptors to promote endogenous tissue regeneration.^[^ ^16^ ^]^ To investigate the cytocompatibility, adhesion and pro‐endothelialization, human umbilical vein endothelial cells (HUVECs) were seeded on PDO, PDGA (gelatin modified PDO), PDAG (A5G81 modified PDO), or PGAG membranes. As shown in **Figure** **3**A, the distribution of HUVECs was sparse and the amount did not obviously increase on the PDO surface from 24 h to 48 h, while the adhesion and proliferation of HUVECs was significantly improved after surface modification, with the maximum count on the PGAG membrane (Figure S8, Supporting Information). The endothelium completely covered the surface of PGAG within 48 h in vitro (Figure 3A). Moreover, the cellular morphology of HUVECs on PDGA and PGAG exhibited elongated and pseudopod‐like structures, while those on PDO and PDAG were significantly smaller and rounder (Figure 3A). The axis ratio of HUVECs on PDGA and PGAG, which indicated the cell morphology of adhesion and extension status, was significantly greater than that on PDO (Figure S9, Supporting Information), indicating the ECM‐mimic structure of gelatin could activate the adhesion and migration of HUVECs. The same phenomenon was also observed in L929 (a rat fibroblast line) implanted on PGAG (Figure S10, Supporting Information), demonstrating that PGAG had the potential to recruit and facilitate proliferation of various cell types in vivo.

To further visualize the adhesion and migration status, cell morphology was inspected by SEM. Figure 3B shows that endotheliocytes on PDO possessed a sphere shape. However, cells tightly sticked and fully extended with multiple pseudopods on the PGAG surface. The coverage area of single cell on PGAG was significantly larger than that of PDO and PDAG (Figure S11, Supporting Information). Collectively, the surface gelatin‐peptide structure on PDO could induce tight adhesion of cells, which was expected to promote the process of endothelium in vivo.

### 2.3 Activation of Integrin‐FAK Related PI3K/AKT and ERK/MAPK Pathways

The molecular mechanism of adhesion and proliferation was further investigated by immunofluorescence staining and western blot. The integrin α3β1 complex is a key regulator for cell migration and re‐epithelialization, and interacts specifically with laminin.^[^ ^15^, ^16^ ^]^ PGAG possess abundant laminin‐derived peptide and ECM similar structure. Therefore, we hypothesized that the cell–material interface would activate the integrin complex to facilitate endotheliocyte bioactivities. As shown in Figure 3C, the integrin α3 was remarkedly activated on PDAG and PGAG membranes compared with PDO, due to the high affinity of A5G81 peptide (Figure S12A, Supporting Information). Focal adhesion kinase (FAK), a cytoplasmic protein tyrosine kinase that distinctly co‐localizes with integrins,^[^ ^23^ ^]^ was also significantly augmented by PDAG and PGAG as a consequence of activation of integrin complex (Figure 3C and Figure S12B, Supporting Information). We further investigated the downstream of FAK, including the pathways of phosphoinositide 3‐kinase/protein Kinase B (PI3K/AKT) and phosphorylated extracellular signal‐regulated kinase/mitogen‑activated protein kinase (Erk/MAPK), which are important in regulating the cell survival, adhesion and proliferation.^[^ ^24^ ^]^ Western blot demonstrated that PI3K/AKT and MAPK pathways were significantly upregulated by stimulus of PDAG and PGAG, consistent with the activation of FAK (Figure 3D–G and Figure S13, Supporting Information). The signaling intensity was greater on A5G81 modified membrane, indicating that the specific affinity of the peptide could additionally activate the survival and proliferation pathway, which was consistent with results of 48 h proliferation assay (Figure 3A). Collectively, these data indicated that endothelial cells could substantially proliferate on PGAG by activation of FAK/PI3K/AKT and Erk/MAPK signaling pathway, due to interactions between integrin receptor and protein–peptide unit (Figure 3H).

### 2.4 Modulation of Inflammation by Decreasing M1 Macrophages and Promoting Proreparative Cytokine Release

**Figure 4:**
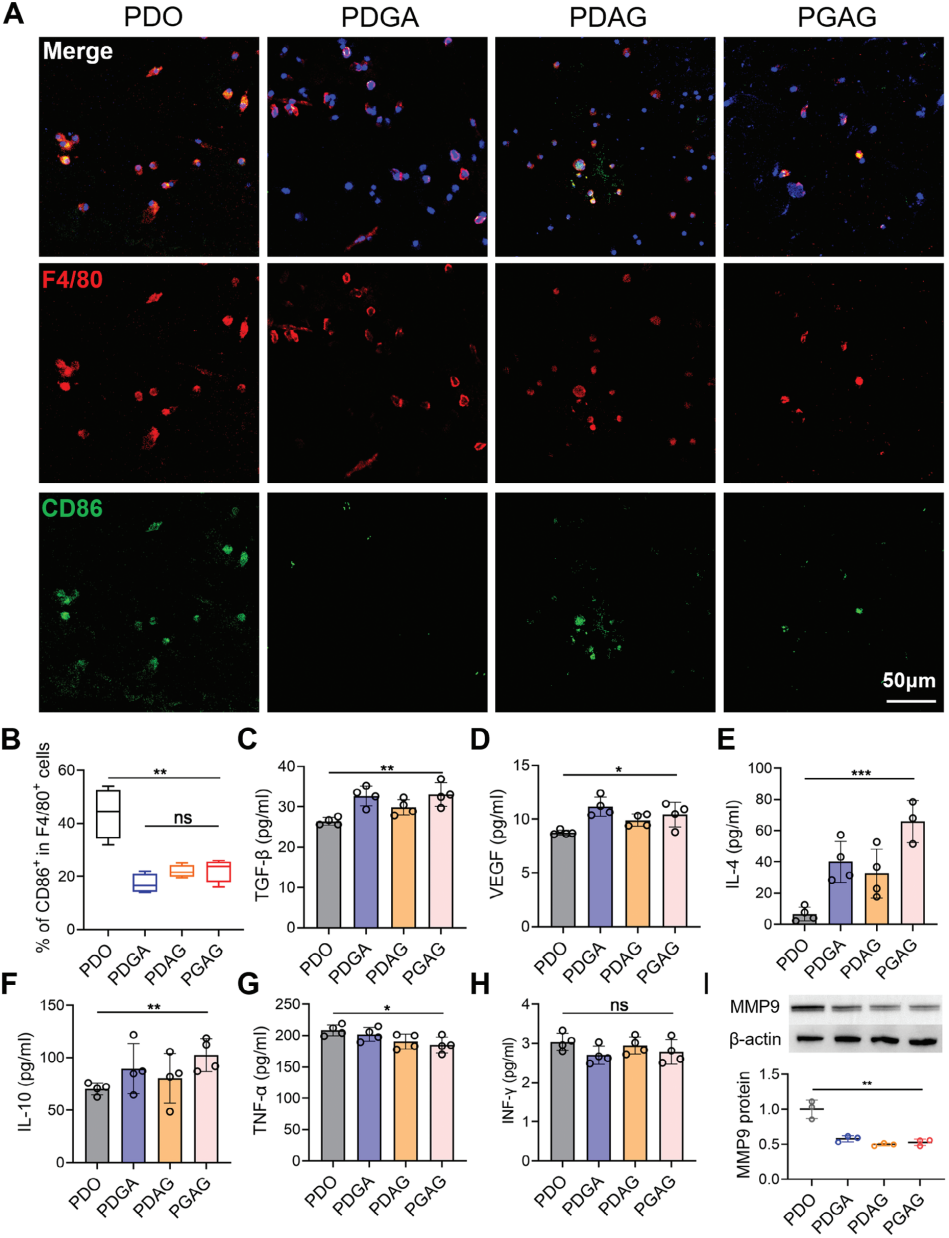
PGAG mitigated inflammation and promoted release of reparative cytokines. A) Representative images of BMDMs treated on PDO, PDGA, PDAG, and PGAG membranes (blue: nuclear; red: F4/80^+^; green: CD86^+^). B) Quantification of percentage of CD86^+^ expression within F4/80^+^ BMDMs (*n* = 3). The box plot indicates the range from min to max. Relative protein expression of C) TGF‐β, D) VEGF, E) IL‐4, F) IL‐10, G) TNF‐α and H) IFN‐γ in supernate of BMDMs determined by ELISA (*n* = 4 for each test). I) Relative protein expression of MMP9 in BMDMs determined by western blotting (*n* = 3). Data are presented as mean ± SD. *p*‐values are calculated using one‐way ANOVA with Bonferroni correction. ns = no significance, *p < 0.05, **p < 0.01, and ***p < 0.001.

Inflammation caused by implanted devices plays a central role in innate tissue regeneration. Immune cells recruited or induced by biomaterials are broadly divided into two categories: pro‐inflammatory (including M1 macrophages) and anti‐inflammatory (such as M2 macrophages).^[^ ^25^ ^]^ Previous studies suggested that the hydrophilia and surface morphology of biomaterials were related to the immune response.^[^ ^26^ ^]^ Gelatin could improve biocompatibility and trigger anti‐inflammation response.^[^ ^22^ ^]^ Thus, we hypothesized that the modification of hydrophilia and biochemical cues from gelatin and peptide could improve biocompatibility by polarizing immune cell toward a pro‐reparative diagram. Bone marrow‐derived macrophages (BMDMs) from C57BL/6 mice were seeded on materials and incubated for 48 h. The morphology was determined by immunofluorescence staining, with F4/80 to label the macrophages and CD86 to identify M1 type. As shown in **Figure** **4**A and Figure S14 (Supporting Information), the number of BMDMs per view did not differ among the four groups, indicating that the interface strategy would not provoke immune cell adhesion and recruitment. However, compared with PDO, the percentage of M1‐type macrophages (F4/80^+^ and CD86^+^) within BMDMs (F4/80^+^) significantly decreased in PDGA, PDAG and PGAG groups (Figure 4A,B), which demonstrated that gelatin and peptide improved the biocompatibility and mitigated the macrophage M1 polarization‐associated inflammatory response.

The expression of representative pro‐reparative cytokines, including transforming growth factor‐β (TGF‐β), vascular endothelial growth factors (VEGF), interleukin‐4 (IL‐4) and interleukin‐10 (IL‐10), and pro‐inflammatory cytokines, such as tumor necrosis factor‐α (TNF‐α) and interferon‐γ (IFN‐γ) were further determined by enzyme‐linked immunosorbent assay (ELISA). Figure 3B–H shows that the concentration of TGF‐β, VEGF, IL‐4 and IL‐10 were substantially up‐regulated by PGAG treatment compared with PDO, while TNF‐α was remarkedly reduced. IFN‐γ was secreted in a relatively low concentration in all groups (mean concentration < 3 pg mL^−1^). The matrix metallopeptidase 9 (MMP9), a key gelatinase linked with inflammation, extracellular matrix degradation and adverse cardiac remodeling,^[^ ^27^ ^]^ was also significantly alleviated by surface modification, which could prevent the constant enzymolysis of the interface and newly formed ECM, consistent with the decreased proportion of pro‐inflammatory macrophages by PGAG treatment. Altogether, these results suggested that PGAG alleviated the inflammatory response and provoked a pro‐reparative process by decreasing M1 macrophages and regulating innate cytokines.

### 2.5 Safety and Efficacy of PGAG Occluder in a Porcine ASD Model

Next, the effectiveness of PGAG occluder in cardiac tissue regeneration was verified in a porcine ASD model with PDO occluder as a control. ASD was created by perforating the atrial septum and subsequent balloon dilatation (Figure S15, Supporting Information). The diameter of created ASD was 8 mm. Therefore, percutaneous transcatheter implantation of PDO and PGAG occluders with a waist diameter of 8 mm and a disc diameter of 16 mm was performed under the transthoracic echocardiography (TTE) and X‐ray guidance. All of the PDO and PGAG occluders were successfully released from the delivery system and occluded the defects at the first attempt. All animals survived in good physical condition after the procedures. No evidence of hematoma, pericardial effusion, valve damage, limb ischemia, dyspnea, or infection occurred during the procedures.

**Figure 5:**
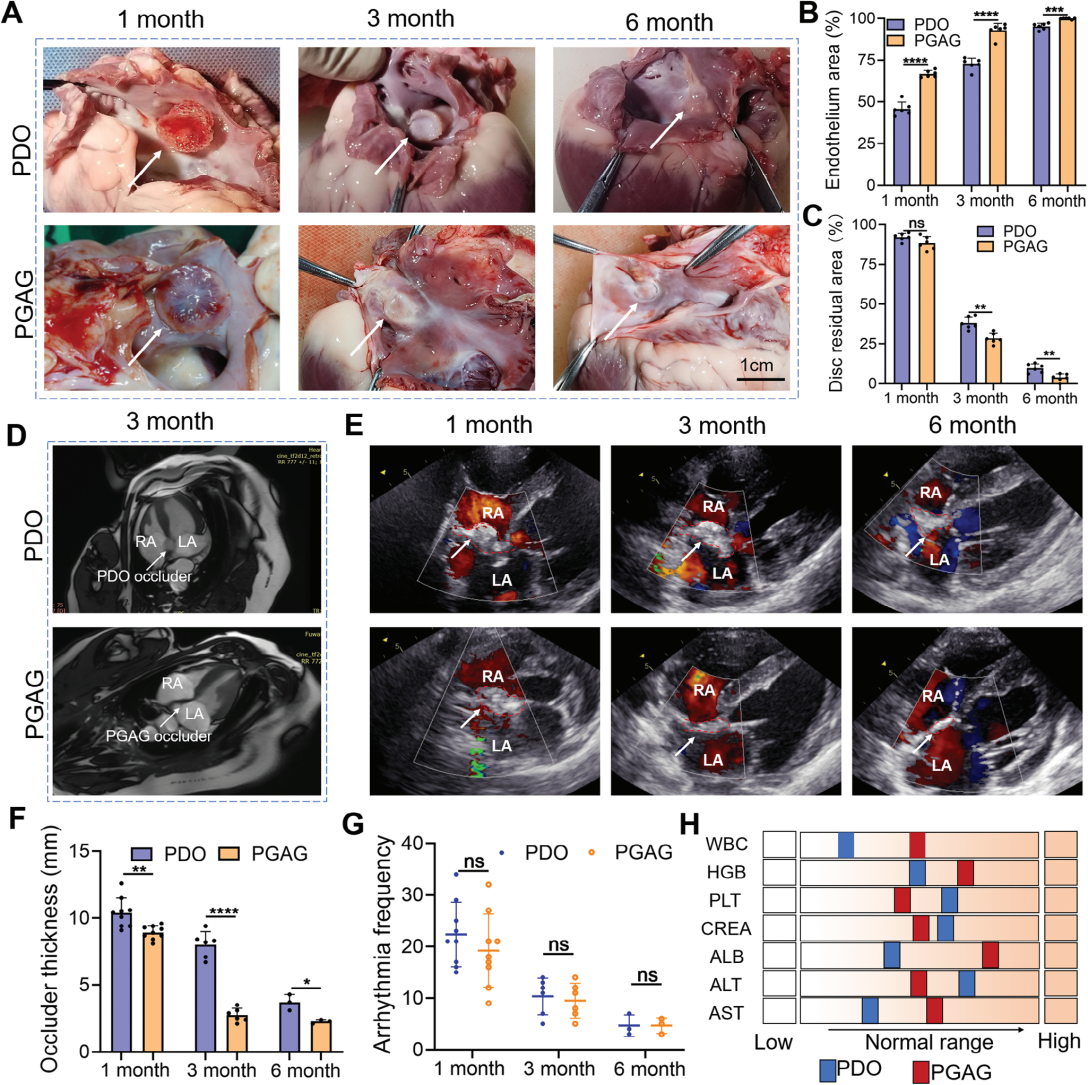
PGAG occluder was safe and effective in a porcine ASD model. A) Macroscopic views of implanted PDO and PGAG occluders in an ASD model at 1, 3, 6 months. B) Quantification of endothelium coverage area of discs (*n* = 3 pigs at each time point, left and right discs for each pig). C) Quantification of disc residual area (*n* = 3 pigs at each time point). D) Cardiac MRI for occluders at 3 months. (E) TTE images of occluders at 1, 3, and 6 months. F) Occluder thickness determined by TTE (*n* = 9 at 1 month, *n* = 6 at 3 month and *n* = 3 at 6 month). G) Frequency of arrhythmia (*n* = 9 at 1 month, *n* = 6 at 3 month and *n* = 3 at 6 month). H) Quantification of blood examination at 6 months. The square indicates the mean value (*n* = 3). WBC: white blood cell; HGB: hemoglobin; PLT: platelet; CREA: creatinine; ALT: albumin; ALT: alanine transaminase; AST: aspartate transaminase. Data are presented as mean ± SD. *p*‐values are calculated using unpaired t test. ns = no significance, *p < 0.05, **p < 0.01, ***p < 0.001, ****p < 0.0001.

At 1, 3, 6 months, pigs were sacrificed to evaluate the efficacy of occlusion. As shown in **Figure** **5**A, all the occluders were well positioned and no displacement happened. The discs of PGAG occluder tightly adhered to the atrial septum and formed a cohesive plane at 1 month, with endothelial coverage over 60% (Figure 5B). However, the discs of PDO occluder were visibly separable from the atrial septum, with 40% overlay by an endothelial layer (Figure 5A,B). At 3 months, the PGAG occluder seamlessly integrated with the surrounding cardiac tissue devoid of any protrusion. While the endothelium covered over 70% of PDO occluder's surface, a discernable cavity persisted between PDO occluder and tissue, and the disc residual area was significantly larger in PDO occluder (Figure 5A,B). At 6 months, the endothelium covered over 95% in both groups (Figure 5B). In PGAG group, the surface of neo‐regenerated tissue appeared smooth, and the PGAG occluder became nearly imperceptible, leaving only a circular boundary. In contrast, the PDO occluder exhibited an uneven surface with a transparent fibrous capsule with significantly more disc residual area (Figure 5A,B). No obvious erosion or destruction of surrounding cardiac tissue and valves occurred in both groups. These anatomic examinations demonstrated that PGAG with bioactive interface could accelerate the process of endothelial coverage, and orchestrate a proper endogenous tissue regeneration.

Multiple imaging methods were utilized during the follow‐up for in vivo functional evaluation. At 3 months, cardiac magnetic resonance imaging (MRI) demonstrated that the neo‐tissue grew into the PGAG occluder and formed an integral septum at 3 months, while the interspace in the PDO occluder was still obvious and a round and protruding fibrous capsule was formed (Figure 5D). TTE showed that the occluder was in the correct position and degraded gradually with time prolonged (Figure 5E). No residual shunt was observed surrounding the occluder in both groups (Figure 5E). However, the occluder and neo‐tissue thickness was significantly higher in PDO group than these in PGAG group, with an obvious disc protrusion at 1 and 3 months (Figure 5F). Cardiac function was in the normal range during the follow‐up (Figure S16, Supporting Information). No valvular stenosis or regurgitation was found.

Arrhythmia is a common but severe complication caused by percutaneous occluder, which is associated with foreign body response and persistent inflammation.^[^ ^28^ ^]^ To assess the safety of biodegradable occluder, arrhythmias were observed by Holter (a dynamic 24 h ECG) during the follow‐up. Figure 5G showed that the frequency declined in accordance with the degradation of occluders in both groups. The occurrence of frequencies was all accidental and transient. The arrhythmia was mainly atrial and ventricular premature beats and no fatal arrhythmia was observed in both groups. The arrhythmia frequency was less in PGAG group at 1 month, but no significant difference was observed between two groups, which could be attributed to the relatively small sample number and short examination period in the animal study. Additionally, the artificial ASD was created relatively far away from the conduction system (for animal safety) compared with natural ASD and VSD, resulting in less disturbance caused by occluders.

No abnormalities were found in both blood tests and hepatic examinations, indicating that PGAG occluder was not toxic during long‐term follow‐up (Figure 5H). No thrombus, infarction or PDO residuals were observed in other organs (liver, spleen, lung, and kidney) in gross and microscopic examination (Figure S17, Supporting Information).

### 2.6 Mitigation of Fibrosis and Inflammation by PGAG Occluder

**Figure 6:**
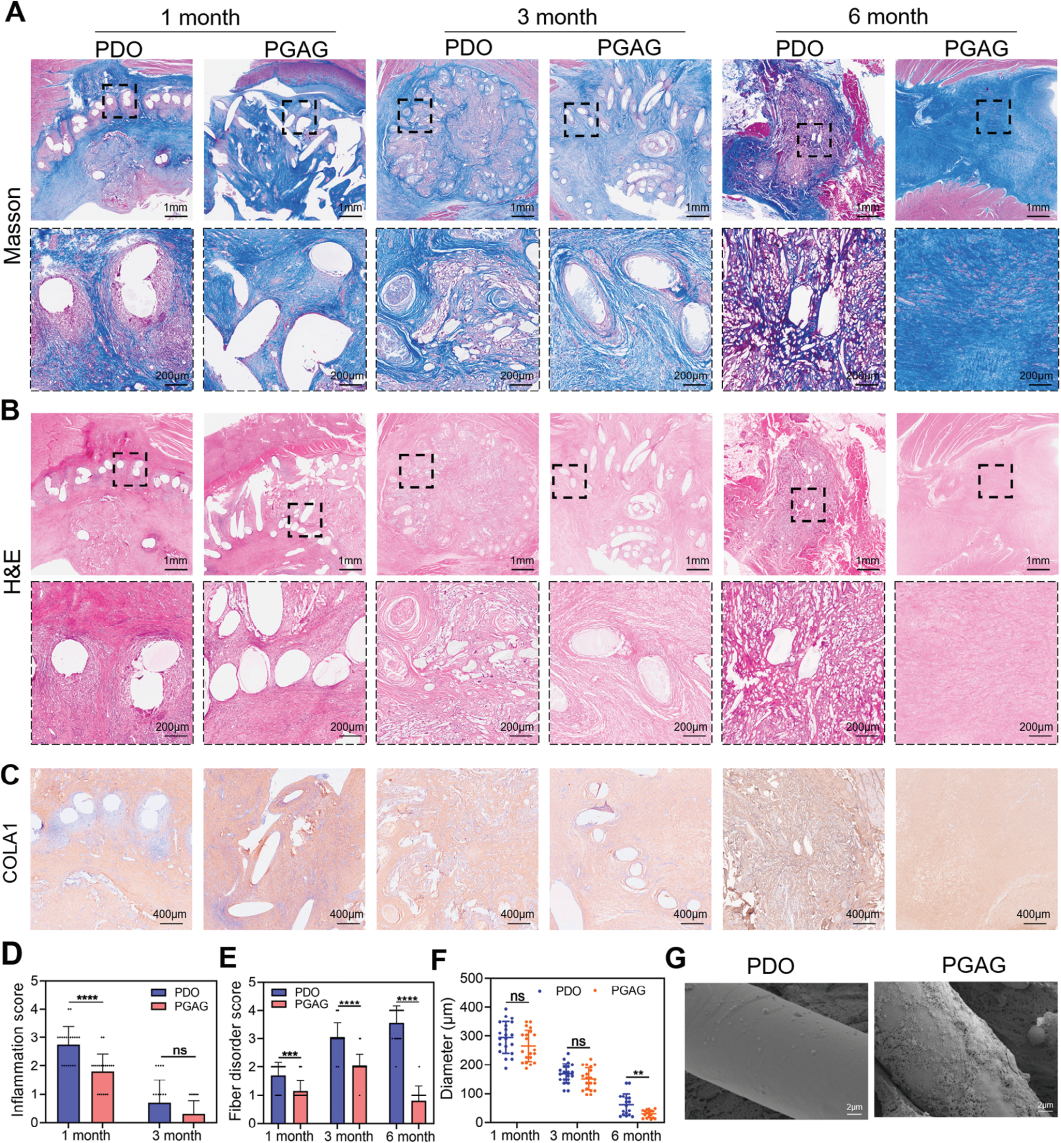
PGAG occluder alleviated inflammation and fibrosis during the regeneration process. A,B) Masson and H&E staining of cardiac tissue surrounding occluders. C) Immunohistochemical staining of COLA1. D,E) Inflammation score and fiber disorder score of surrounding fibers, with score 0 = normal, 1 = slight, 2 = mild, 3 = moderate, 4 = severe (*n* = 20 at each time point). F) Diameter of monofilament (*n* = 20 at each time point). **G**) Representative SEM image of monofilament at 1 month. Data are presented as mean ± SD. *p*‐values are calculated using unpaired t test. ns = no significance, *p < 0.05, **p < 0.01, ***p < 0.001, ****p < 0.0001.

Masson's trichrome and H&E staining were performed to analyze the pathological changes. Masson's trichrome staining shows that the newly formed collagen was distributed disorderly in the PDO group, while the fiber arrangement was ordered and tight in the PGAG group (**Figure** **6**A). Furthermore, excessive collagen disposition formed a fibrous capsule besieging the PDO fibers at 1, 3, and 6 months, while it was unobservable surrounding the PGAG fibers (Figure 6A). H&E staining showed that the area of fibrous capsule accompanied with moderate inflammatory response indicated by immune cell infiltration at 1 month, while mild inflammation was observed by PGAG treatment (Figure 6B). Notably, the infiltration of inflammatory cell was also found in the atrial septum tissue by the PDO treatment at 1 month, which might cause tissue destruction and affect the conduction system (Figure 6B). The inflammation score of cardiac tissue was significantly higher in PDO group and reached a moderate inflammation degree at 1 month, while it was mild degree in PGAG group (Figure 6D). At 3 months, the inflammation score decreased to slight degree in both groups (Figure 6D). At 6 months, the inflammation in PGAG group was completely degraded and constructed a compact and ordered collagen barrier between the cardiac tissue, while the collagen was organized in a loosened and irregular array by PDO treatment, owing to the moderate and repetitive inflammatory response induced by PDO from 1 to 3 months (Figure 6A,B).

To further evaluate the degree of fibrosis, the expression of COLA1, a collagen formed in the scar tissue, was determined by immunohistochemistry. In accordance with Masson staining, the arrangement of COLA1 was more compact and regularly ordered surrounding PGAG fibers, compared with PDO (Figure 6C). The fiber disorder score was significantly higher in PDO group and increased from 1 to 6 months (Figure 6E), while the score decreased to mild degree at 6 months in PGAG group. Figure 6F shows that the diameter of PDO and PGAG fibers decreased evenly from 1 to 6 months, suggesting the steady degradation process. No significant difference was observed between the two groups at 1 and 3 months, indicating that the interface did not affect the degradation of PDO fibers. At 6 months, most polymers in both groups degraded (Figure 6A,B). The diameter of remaining polymers was slightly higher in PDO group (29.4 ± 13.3 µm in PGAG vs 61.5 ± 38.0 µm in PDO, p = 0.0025), which could be attributed to the influence of fibrous encapsulation (Figure 5A). Representative images of SEM showed that a fibrillar layer similar to natural cardiac ECM formed on the surface of PGAG at 1 month and multiple cells with pseudopodium tightly adhered (Figure 6G). However, cells with round morphography and small size sparsely distributed on the bare PDO (Figure 6G). All these results indicated that the PGAG occluder treatment orchestrated a well‐organized endogenous regeneration without excessive inflammation and fibrosis formation.

### 2.7 Promotion of Endothelialization and Regulation of Immune Response by PGAG Occluder

**Figure 7:**
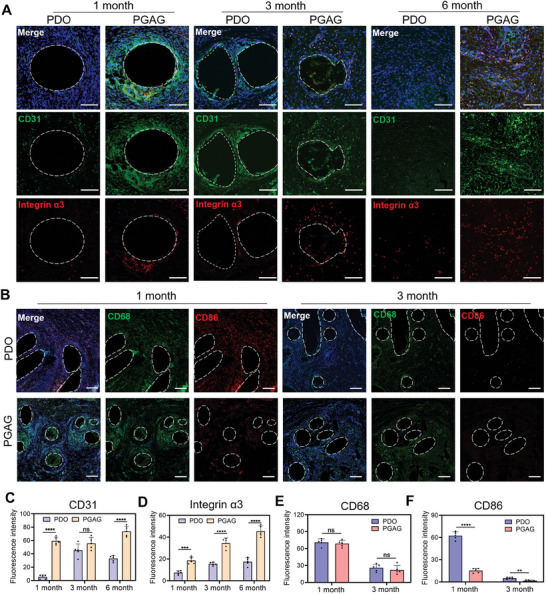
PGAG occluder promoted endothelialization and mitigated polarization of pro‐inflammatory macrophages. A) Immunofluorescence staining of CD31 (green) and integrin α3 (red). The white line indicates 100 µm. B) Immunofluorescence staining of CD68 (green) and CD86 (red). The white line indicates 200 µm. Statistical data of average fluorescence intensity of C) CD31, D) integrin α3, E) CD68, and F) CD86 (*n* = 5 for each test). Data are presented as mean ± SD. *p*‐values are calculated using unpaired t test. ns = no significance, *p < 0.05, **p < 0.01, ***p < 0.001, ****p < 0.0001.

Endothelium process was further evaluated by immunofluorescence staining of CD31, a marker of endotheliocytes. As shown in **Figure** **7**A, the number of endotheliocytes surrounding the PGAG fiber was significantly higher than the PDO fiber at 1 month (Figure 7A,C). What is more, the integrin α3, which related to the interaction with the peptide interface, was remarkedly up‐regulated by PGAG treatment (Figure 7A,D), indicating the activation of cell survival, adhesion, and proliferation. Both PDO and PGAG triggered endothelial cell encapsulation at 3 months, while PGAG still induced more integrin protein expression (Figure 7C,D). At 6 months, the endotheliocyte and integrin receptor remained at a significantly higher intensity in PGAG group than PDO group after the fiber degraded, which indicated that the activation of endogenous regeneration process was persistent and the repair process was dominated by endothelium (Figure 7A,C,D). The macrophages, indicated by CD68 positive staining, were obviously recruited around PDO and PGAG fibers at 1 months. However, the percentage of pro‐inflammatory type of macrophages was significantly lower in PGAG group (Figure 7B,E,F). Meanwhile, the number of surrounding macrophages decreased significantly in both groups from 1 to 3 months, and the pro‐inflammatory type was almost invisible in PGAG group at 3 months (Figure 7B,F). Collectively, the process of endothelialization was activated and accelerated, and the inflammation response was down‐regulated by PAGA occluder during ASD therapy.

### 2.8 Immunomodulation and Proendothelialization in Endogenous Tissue Regeneration Revealed by Multiomics

**Figure 8:**
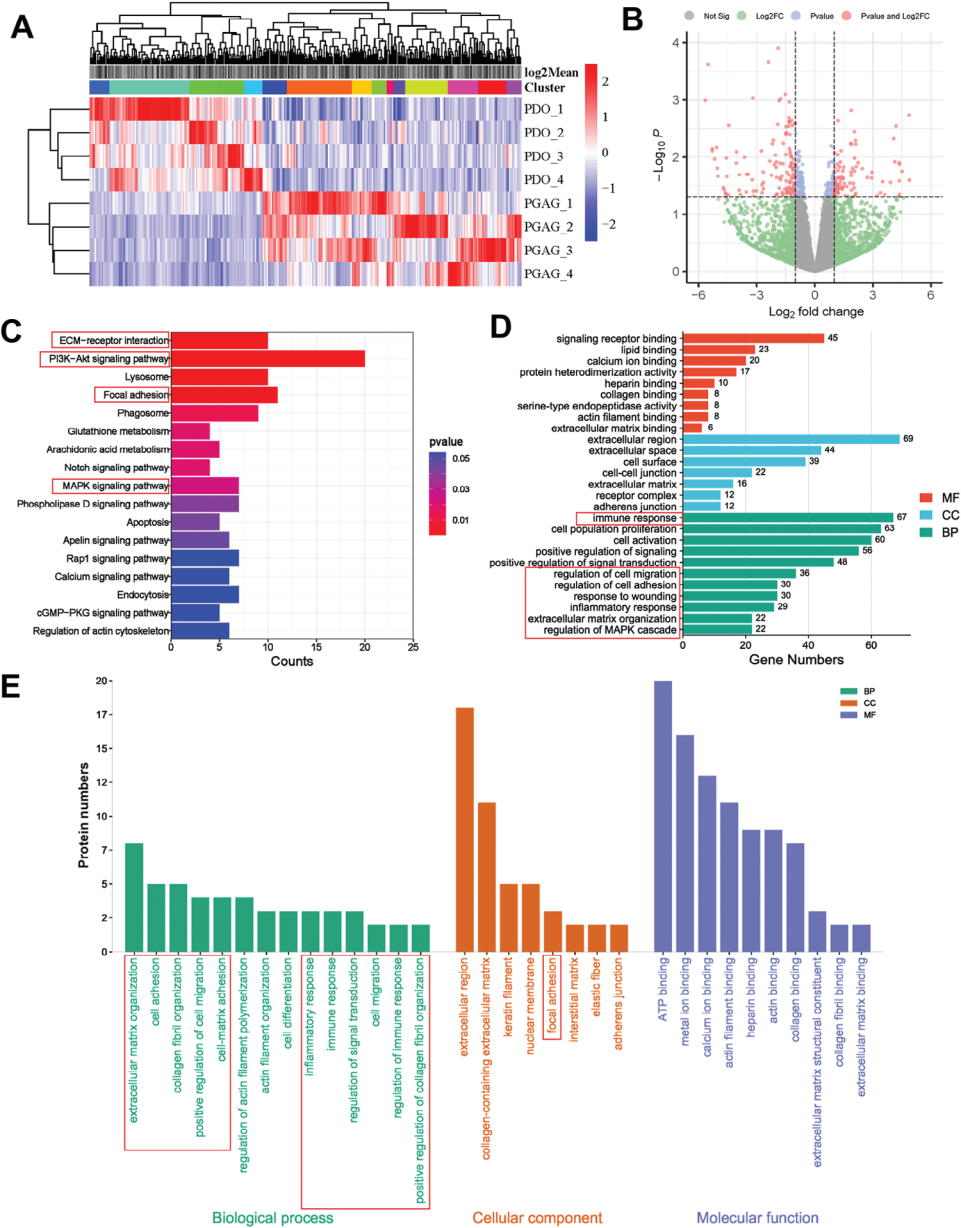
Immunomodulation and proendothelialization revealed by multiomics. A) Cluster heatmap of significantly upregulated and downregulated DEGs by transcriptomics. B) Volcano plots. The red dots indicate the proteins selected based on adjusted *P* value < 0.05 and log_2_FC > 2. C) KEGG pathway‐enrichment analysis of DEGs. D) GO pathway‐enrichment analysis of DEGs. E) GO pathway‐enrichment analysis of DEPs by proteomics. BP: biological process; CC: cellular component; MF: molecular function.

Finally, we performed multi‐omics of neo‐formed tissue at 3 months to comprehensively investigate the mechanisms of PGAG induced tissue regeneration. Transcriptomics revealed that a total of 324 differentially expressed genes (DEGs) including 130 genes upregulated and 194 genes downregulated were identified in transcriptomics (**Figure** **8**A). The DEGs were visualized by the cluster heatmap (Figure 8B). The top significantly enriched terms in KEGG pathway enrichment comprised ECM–receptor interaction, PI3K‐AKT signaling pathway, focal adhesion, and MAPK signaling pathway (Figure 8C), which was consistent with the cellular signaling investigation. GO pathway‐enrichment analyses including biological process (BP), cellular component (CC), and molecular function (MF) were conducted to demonstrate the main biological processes regulated by PGAG (Figure 8D). MF analysis included signaling receptor binding, collagen binding, actin filament binding and ECM binding. CC analysis mainly referred to extracellular region, cell–cell junction, adherens junction, indicating the interaction between cell and ECM–mimic interface. MF analysis revealed that immune response, cell population proliferation, cell activation, regulation of cell migration, regulation of cell adhesion, response to wounding, ECM organization and regulation of MAPK cascade were significantly modulated by PGAG treatment. To further testified these findings of transcriptomics, proteomics was also performed. A total of 2,299 proteins were identified, including 132 up‐regulated and 61 down‐regulated differentially expressed proteins (DEPs) (Figure S18, Supporting Information). GO analysis demonstrated that ECM organization, cell adhesion, collagen fibril organization, cell‐matrix adhesion, cell differentiation, regulation of immune response, collagen biding, and ECM binding pathways were significantly enhanced by PGAG treatment (Figure 8E). Both the transcriptomics and the proteomics corroborated each other and coincided with the in vitro investigation, further confirming that PGAG occluder could program multiple cell survival, adhesion, and migration, as well as immunoregulation pathways for endogenous tissue regeneration.

## 3 Summary and Discussion

In summary, we have demonstrated the efficacy of a biophysically and biochemically optimized biodegradable cardiac implant for promoting in situ tissue regeneration in a porcine ASD model. A bioactive polymer with a structure composed of gelatin and laminin‐derived A5G81 peptide through covalent bonds formed by Michael addition reaction was modified on the surface of the implant material. The bioactive cell‐material interface can significantly promote endotheliocyte adhesion and proliferation by activation of integrin receptor and FAK complex. Moreover, the interface improved the biocompatibility of the implantation by polarizing immune cells into anti‐inflammatory phenotypes and augmenting the release of reparative cytokines. In vivo implantation of the PGAG occluder in pigs showed activation of endothelialization and suppression of inflammatory responses and excessive fibrosis in ASDs. In situ tissue regeneration was mainly attributed to significant enhancement of ECM remodeling, endogenous cell adhesion, cell proliferation, immune response regulation, and activation of ECM binding pathways by PGAG treatment.

Enhancing the rapid endothelialization and facilitating tissue regenerative processes are pivotal considerations in the design of biodegradable occluders, crucial for the success of occlusion, prevention of displacement, and inhibition of thrombosis. Various surface modifications, utilizing ECM derived or ECM mimic polymers such as chitosan, collagen, elastin, gelatin, laminin, and decellularized ECM tissue, have been extensively explored to enhance the physiochemical and biological characteristics of cardiovascular implants.^[^ ^29^ ^]^ Gelatin, an ECM derivative from collagen, stands out for its ability to mimic native vascular architecture, thereby improving biocompatibility and enhancing implant hydrophilicity, which, in turn, facilitates cell adhesion and proliferation while inhibiting thrombosis.^[^ ^30^ ^]^ However, gelatin modification alone lacks the specificity needed for recruiting and proliferating endothelial cells, making it less effective in promoting endothelialization compared to coatings with specific antibodies like CD34 or CD133.^[^ ^31^ ^]^ Nevertheless, specific antibody or drug coatings often have a short in vivo lifespan and are impractical for clinical storage. As an alternative, endotheliocyte‐selective peptides, such as A5G81, present a promising avenue for specifically amplifying endothelial cell recruitment.^[^ ^32^ ^]^ Yet, peptides have limitations in terms of spatial architecture and stability, rendering them less suitable for the migration, spread, and proliferation of various cell types in vivo. This study introduces a grafting modification strategy to augment cell‐material interactions and promote endogenous tissue regeneration for cardiac implants. The gelatin‐peptide modification serves a dual purpose by providing ECM‐derived bioactive cues and serving as a rough, hydrophilic substrate for cell adhesion and proliferation. Simultaneously, the endotheliocyte‐selective peptide (A5G81) is incorporated to activate proliferative signaling pathway and enhance endothelialization. This innovative approach holds promise for advancing the field of biodegradable cardiovascular implant design, addressing limitations associated with present biodegradable cardiac occluder. The simple and economical manufacture process and storage condition would facilitate the clinical translation.

Our study still remained some limitations for future work. First, although porcine ASD models are standardized animal models for investigations of CHDs and occluder implantation, the location and size of artificial ASDs cannot adequately reflect the conditions of the ASD patients, which may underestimate the complications such as arrhythmia, residual shunt or cardiac erosion. Second, this surface modification strategy may also benefit interactions with other cell populations participating in endogenous tissue repair such as platelets, T cells and fibroblasts, which calls for further investigations to comprehensively unveil the proregenerative mechanisms. Third, multiple time points of multi‐omics analysis, e.g., <1 month, would allow for a horizontal and longitudinal contrast, and offer a comprehensive understanding of immune response, population dynamics, and the impact of mitigating inflammation during the early stages of endogenous tissue regeneration on the bioactive material. Finally, the storage condition and the effective time of the interface should be further evaluated for clinical use. Collectively, the PGAG occluder offers a simple and economical manufacture process to facilitate cardiac defect repair, which we envision could provide clinical opportunity to improve the prognosis for CHD patients.

## 4 Experimental Section

### Chemical Reagents

(3‐Glycidoxypropyl) trimethoxy silane (GOPS) and gelatin were purchased from Sigma‐Aldrich. Mal‐A5G81 (Mal‐AGQWHRVSVRWGC) was provided by Bankpeptide Biological Technology Co., LTD (Hefei, China). PDO occluders were provided by Shanghai Shape Memory Alloy Co. Ltd.

### Synthesis of Gelatin‐A5G81

Gelatin‐A5G81 was synthesized by mixing gelatin and Mal‐A5G81 (1:2 in molar ratio) in aqueous solution for 3 h at room temperature, and further dialyzed against water and lyophilized. Mal‐A5G81 peptide was bonded to gelatin molecule through the amino‐maleimide click reaction.

### Preparation of PGAG Occluder

PDO occluder is prepared through three steps. First, PDO occluder was placed in the plasma surface treatment machine, and pure O_2_ was fed in, and the excitation frequency of the plasma surface treatment machine was set to 13.56 MHz, and the working time was 120 s. Subsequently, PDO occluder (≈170 mg) treated with oxygen plasma was immediately immersed in 50 mL of 1% GOPS aqueous solution, and reacted at room temperature for 120 min, so that reactive epoxy groups were bonded to the surface of PDO occluder. Finally, the PDO occluder treated in the second step was immersed in 50 mL of 0.5% gelatin‐A5G81 aqueous solution, and continued to react at room temperature for 12 h, and finally gelatin‐A5G81 was bonded on the surface of the occluder material. The amino groups on the gelatin react with the epoxy groups on the surface of the PDO occluder to achieve gelatin‐A5G81 bonding. PDGA and PDAG was prepared by surface plasma treatment of PDO and modified with silane coupling agent, and further covalently grafted with gelatin and A5G81, respectively.

### Evaluation of Cytocompatibility, Adhesion, and Proliferation of HUVECs

To evaluate the cytocompatibility, adhesion and proliferation, PDO, PDGA, PDAG, PGAG membranes with thickness of 40 µm were prepared. Then, the membranes were plated in 6‐well plates. A total of 1.5 × 10^5^ HUVECs were seeded on each membrane, and were cultured in endothelial cell medium supplied with 10% FBS. After incubation for 24 h and 48 h, the membranes were washed with PBS for 3 times, stained with crystal violet solution (0.5%) for 30 min and the cells on membranes were counted.

For immunofluorescent staining, after incubation for 24 h, the cells seeded on membranes were incubated with Integrin α3 (Proteintech, 66070‐1) and FAK (Abcam, ab40794) overnight, and were stained with 488 conjugate (Cell Signaling, #4408), 594 conjugate (Cell Signaling, #8889) and DAPI (Solarbio, C0065) according to the manufacture's guidelines and were observed by confocal laser scanning microscopy (TCS SP5II, Leica, Ernst‐Leitz‐Strasse, Germany).

The interior morphology of HUVECs on PDO, PDGA, PDAG, and PGAG membranes was investigated by SEM (S‐4800, Hitachi, Japan). Samples were fixed in 2.5% glutaraldehyde, dehydrated by CO2 critical point drying (K850X, Emitech), quick‐frozen in liquid nitrogen, lyophilized, and then coated with gold particles.

### Western Blot Assays

Cell lysates were lysed by RIPA lysis buffer (Beyotime, P0013B) containing 1 × 10^−3^ m PMSF (Beyotime, ST506). After incubation for 30 min on ice, the supernatant was collected after centrifugation. Then protein was denatured at 95 °C for 10 min. After mixing with loading buffer containing bromophenol blue, protein samples were separated by a 10% sodium dodecyl sulfate‐polyacrylamide gel electrophoresis (SDS‐PAGE) and transferred onto poly (vinylidene difluoride) (PVDF) membranes (0.45 µm). The membranes were blocked by bovine albumin and then incubated with antibody overnight at 4 °C. After washing with TBST, the membranes were incubated with horseradish peroxidase‐conjugated secondary antibodies for 1 h at room temperature. The proteins on the membranes were visualized by Chemiluminescence Imaging system (ChemiScope 6000 Pro, China). The intensity of immunoreactive bands was quantified by ImageJ software. The primary antibody included FAK (1:1000, Abcam, ab40794), phospho‐MEK1/2 (Ser217/221) (1:1000, Cell Signaling, #9154), p44/42 MAPK (Erk1/2) (1:1000, Cell Signaling, # 4695), PI3 Kinase p110a (C73F8) (1:1000, Cell Signaling, #4249), and Phospho‐Akt (Ser473) (1:1000, Cell Signaling, #4060) and β‐actin (1:2000, Abcam, ab8226). The secondary antibody included HRP‐labeled goat anti‐mouse IgG (1:2000, Beyotime, A0216) and HRP‐labeled goat anti‐rabbit IgG (1:2000, Beyotime, A0208).

### Macrophage Polarization

BMDMs were isolated from C57BL/6 mice (6 weeks old, Vital River Laboratory, China). After lysing red blood cells, the collected cells were seeded in six‐well plates and cultured with RPMI 1640 medium supplemented with 10% heat‐inactivated fetal bovine serum and 20 ng mL^−1^ M‐CSF (MCE, HY‐P7085). After incubation for 6 d, adhered cells were BMDMs. After that, BMDMs were seeded on PDO, PDGA, PDAG, PGAG membranes, cocultured for 48 h, washed with PBS for 3 times, and stained with FITC‐labeled anti‐CD86 antibodies (Biolegend, 105006), PE‐labeled F4/80 antibodies (Biolegend, 123110) and DAPI (Solarbio, C0065) according to the manufacture's guidelines and were observed by confocal laser scanning microscopy (TCS SP5II, Leica, Ernst‐Leitz‐Strasse, Germany).

### ELISA

The supernatant of BMDMs was analyzed by Mouse TGF‐β1 Precoated ELISA kit (DAKEWE, 1217102), Mouse IL‐4 Precoated ELISA kit (DAKEWE, 1210402), Mouse IFN‐γ Precoated ELISA kit (DAKEWE, 1210002), Mouse TNF‐α ELISA KIT (CUSABIO, CSB‐E04741m), Mouse VEGF ELISA Kit (CUSABIO, CSB‐E04756m), Mouse IL‐10 ELISA KIT (SenBeiJia Biological Technology Co., Ltd., SBJ‐M0073) according to the manufacturer's instructions.

### Transcatheter ASD Closure of Porcine Model

All animal experiments were approved by the Institutional Animal Care and Use Committee, Fuwai Hospital, Chinese Academy of Medical Sciences (0101‐1‐18‐ZX(X)2). A total of 18 Bama mini‐pigs (male, 30–40 kg) were implanted with the PDO and PGAG occluder through transcatheter access (*n* = 9 each group). Specifically, after general anesthesia and tracheal intubation, all pigs were fixed on the operating table in a supine position. ECG, heart rate, blood pressure, and blood oxygen saturation were monitored during the operation. The right femoral vein was punctured. The guide wire was inserted into the right atrium under the guidance of the fluoroscopy. Then, a septal puncture needle was used to puncture the fossa ovalis. An 8 mm diameter balloon catheter was used to create an ASD model. After that, transcatheter ASD occlusion was implanted under the TTE and fluoroscopy guidance. Ampicillin (1 g) was administered intravenously after implantation and all pigs received aspirin (5 mg/kg/d) postoperatively for 3 d.

Animals were followed up at 1, 3, and 6 months (*n* = 3 each group at each time point). TTE, 24 h‐ dynamic electrocardiogram (Holter monitor) and hematological tests were performed at each point. The diameters of the left and right discs of the occluder were measured at each time point. General anatomical examination, H&E staining, Masson staining, and SEM were performed for analysis. Inflammation was blindly graded by a pathologist using an inflammatory score (0‐4+ scale), with 4+ representing maximal inflammation.^[^ ^33^ ^]^

### Immunofluorescence and Immunohistochemistry for Porcine Tissue

The neo‐formed cardiac tissue surrounding occluders were harvested and fixed with 4% paraformaldehyde, embedded in paraffin, and sliced into 4 µm thick sections. For macrophage polarization investigation, the slices were incubated with mouse CD68 monoclonal antibody (1:200, Proteintech, 66231‐2‐Ig) and rabbit CD86 monoclonal antibody (1:200, Cell Signaling, #76755). For endothelialization analysis, the slices were incubated with mouse Integrin α3 (Proteintech, 66070‐1) and rabbit CD31 Polyclonal antibody (1:200, Proteintech, 11265‐1‐AP). After incubation overnight, slices were incubated with Alexa Flour 488 anti‐mouse IgG (1:200, Thermo Fisher Scientific), Alexa Flour 594 anti‐rabbit IgG (1:200, Thermo Fisher Scientific), or anti‐rat Alexa Fluor 488 Conjugate (Cell Signaling, #4416) and anti‐mouse Alexa Fluor 594 Conjugate (Cell Signaling, #8890) corresponding to the primary antibody. Images were observed by CLSM and quantitatively analyzed by ImageJ software.

For immunohistochemistry assay, the slices were incubated with COL1A1 (1:200, Cell Signaling, #72026) overnight and enhanced enzyme labeled goat anti‐mouse/rabbit IgG polymer (ZSGB‐BIO, PV‐6000) at room temperature for 20 min. Fiber disorder score, judged by Masson staining and immunohistochemistry of COL1A1, was blindly graded by a pathologist using a 0–4+ scale, with 4+ representing maximal disorder (Figure S19, Supporting Information).

### Transcriptomics and Proteomics Analysis

At 3 months, cardiac neo‐tissue from PDO and PGAG treatment was collected (4 samples for each group). For transcriptome, total RNA in cardiac tissue was extracted and the concentration and purity were measured by NanoDrop 2000 Spectrophotometer (Thermo Fisher Scientific, Bremen, Germany). Illumina platform was used to construct a sequencing library and perform PE150 sequencing. Similarly, for proteomics, Nanoflow LC MS/MS analysis of tryptic peptides was conducted on a quadrupole Orbitrap mass spectrometer (Q Exactive HF X, Thermo Fisher Scientific, Bremen, Germany) coupled to an EASY nLC 1200 ultra high‐pressure system (Thermo Fisher Scientific) via a nanoelectrospray ion source. All RAW files were analyzed using the Proteome Discoverer suite (version 2.4, Thermo Fisher Scientific). The functional analysis of RNA and proteins was further analyzed by the Gene Ontology (GO) database and the KEGG (Kyoto Encyclopedia of Genes and Genomes) database. Experiment details are provided in supporting materials.

### Statistical Analysis

Statistical analysis was performed using GraphPad Prism 8 (GraphPad Software). Data were expressed as mean ± SD. Comparisons between two groups were performed with unpaired Student's t‐test. For multiple group comparison, one‐way ANOVA was used with Bonferroni post correction. Statistical significance is denoted by *p < 0.05, **p < 0.01, ***p < 0.001 and ****p < 0.0001.

## Conflict of Interest

The authors declare no conflict of interest.

## Author Contributions

P.K., X.L., and Z.L. contributed equally to this work. Conceptualization: P.K., X.L., W.W., X.P.; Methodology: P.K., X.L., Z.L., R.G., J.W., Z.F., P.H., S.W., F.Z., W.O., W.W., and X.P.; Investigation: P.K., X.L., Z.L., J.W., S.F., R.G., S.W., F.Z., D.Z.; Visualization: P.K., X.L., R.G., J.W.; Funding acquisition: W.W., X.P., W.O.; Project administration: P.K., X.L, Z.L.,W.W., X.P., W.O.; Supervision: W.W., X.P., P.H., Z.F.; Writing—original draft: P.K., X.L.; Writing—review & editing: All authors.

## Supporting information

Supporting Information

## Data Availability

The data that support the findings of this study are available from the corresponding author upon reasonable request.
