# Supplementary material for: Biodegradable Cardiac Occluder with Surface Modification by Gelatin–Peptide Conjugate to Promote Endogenous Tissue Regeneration
Source: Adv Sci (Weinh). 2023 Nov 20;11(2):2305967. doi: 10.1002/advs.202305967 (PMC10787076; doi:10.1002/advs.202305967)
Supplement: Supplementary file 1 — Supporting Information [file ADVS-11-2305967-s001.pdf]

ADVANCED SCIENCE
Open Access

Supporting Information

for *Adv. Sci.*, DOI 10.1002/advs.202305967

Biodegradable Cardiac Occluder with Surface Modification by Gelatin–Peptide Conjugate to Promote Endogenous Tissue Regeneration

Pengxu Kong, Xiang Liu, Zefu Li, Jingrong Wang, Rui Gao, Shuyi Feng, Hang Li, Fengwen Zhang, Zujian Feng, Pingsheng Huang, Shouzheng Wang, Donglin Zhuang, Wenbin Ouyang, Weiwei Wang* and Xiangbin Pan**

Supplementary Materials for

Biodegradable Cardiac Occluder with Surface Modification by Gelatin-Peptide Conjugate to Promote Endogenous Tissue Regeneration

Pengxu Kong, Xiang Liu, Zefu Li, Jingrong Wang, Rui Gao, Shuyi Feng, Hang Li, Fengwen Zhang, Zujian Feng, Pingsheng Huang, Shouzheng Wang, Donglin Zhuang, Wenbin Ouyang, Weiwei Wang*, Xiangbin Pan**

***Corresponding author**

Prof. Xiangbin Pan. Email: panxiangbin@fuwaihospital.org.

Prof. Weiwei Wang. Email: wwwangtj@163.com.

Dr. Wenbin Ouyang. Email: droywb31@163.com.

This file includes:

Methods

Figures S1 to S19

Methods

General characterization information. Fourier transform infrared (FTIR) spectra was performed on a Breaker Alpha spectrometer at room temperature in the range between 4000 cm^{-1} and 400 cm^{-1}.

The amino acid concentration was determined by Amino Acid Assay Kit (R30299, Shanghai yuanye Bio-Technology Co., Ltd, Shanghai, China) according to the instructions.

X-ray photoelectron spectroscopy (XPS) spectra was performed by Thermo Scientific ESCALab250xi, and the profiles analyzed using Avantage software system.

Differential scanning calorimeter (DSC) measurements were performed using TA Instruments DSC Q2000 with a refrigerated cooling system operating from -50 ^{o}C to 125 ^{o}C at a scanning rate of 5 $^{o}C \cdot min^{-1}$ under N_2 atmosphere. The DSC data of elastomeric samples are derived from the second heating curve and the first cooling curve.

Gel permeation chromatography (GPC) measurements were performed using Agilent PL-GPC220 with DMF as the mobile phase.

Static water contact angle of PGAG surface was performed by Dataphysics OCA40.

Mechanical test. PGAG frame sample mechanical performance test was performed using Instron-3400 (34SC-1), and the strain rate 10 $mm \cdot min^{-1}$ was adopted. The initial distance between the two clamps on the Instron machine was 10 mm. The test was repeated at least three times and the average values were recorded.

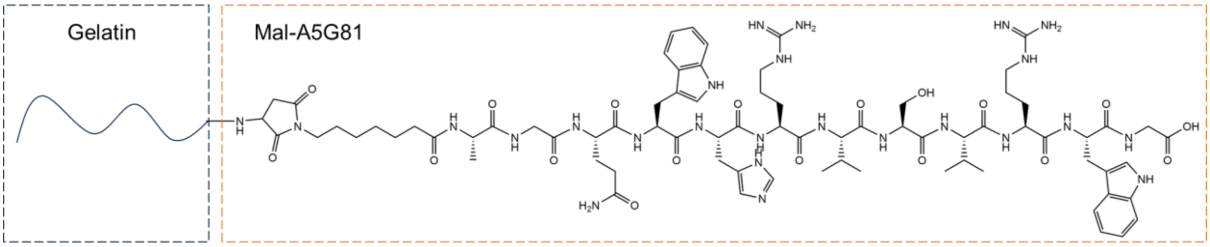

Figure S1. Presentative presentation of gelatin-A5G81 polymer.

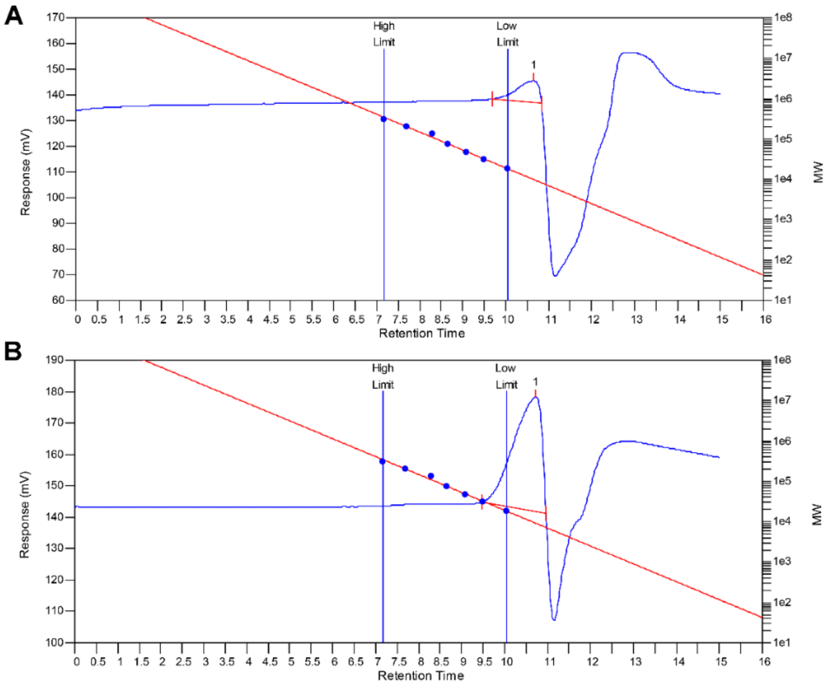

Figure S2. GPC traces for gelatin-A5G81(A) and gelatin (B).

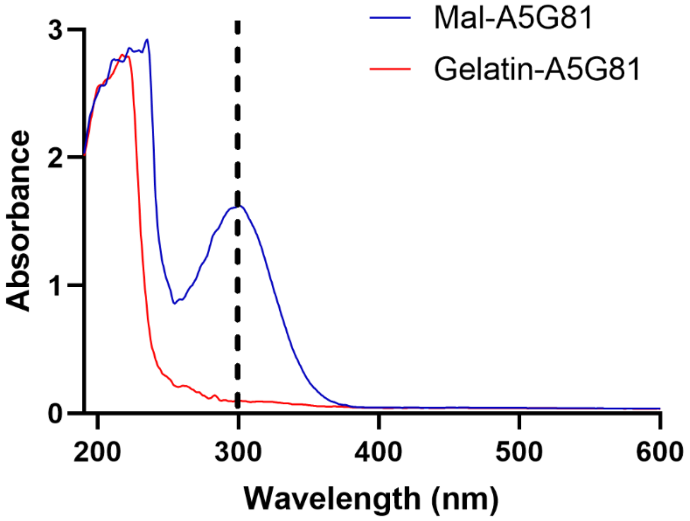

Figure S3. The UV–vis spectrum of Mal-A5G81 and gelatin-A5G81.

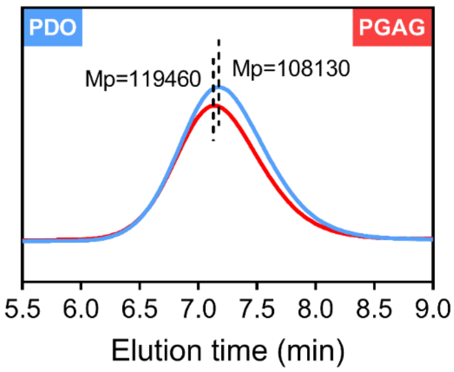

Figure S4. GPC traces for PGAG and PDO.

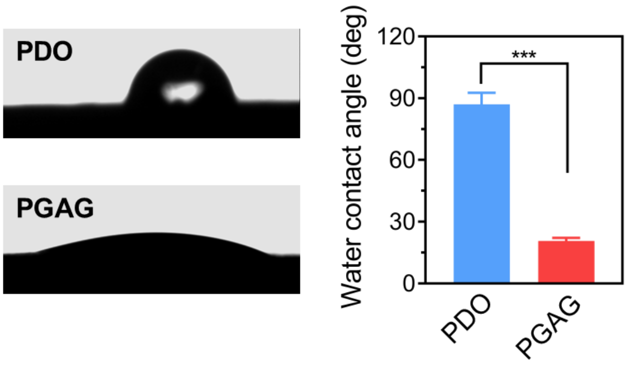

Figure S5. The water contact angle of PGAG (n = 3). Data are presented as mean ± SD. p-values are calculated using unpaired t test. ***$p < 0.001$.

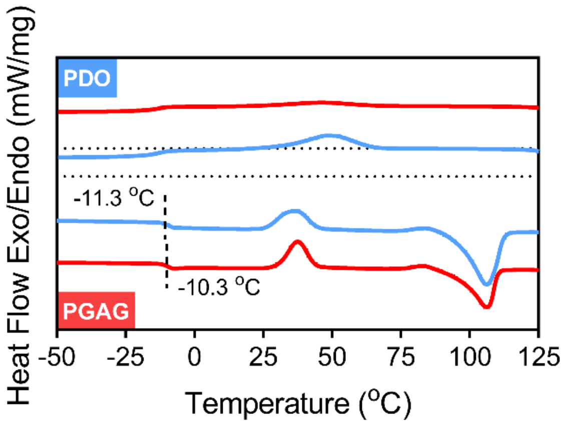

Figure S6. The DSC curves of PGAG material were recorded during the first cooling process and the second heating process.

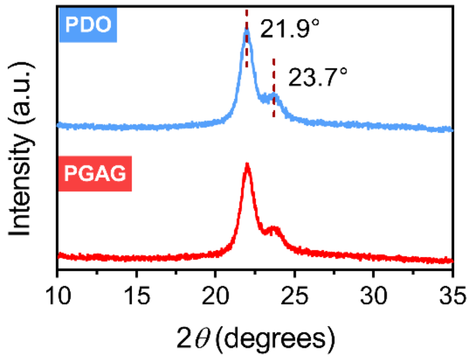

Figure S7. XRD results for monofilament material of PGAG.

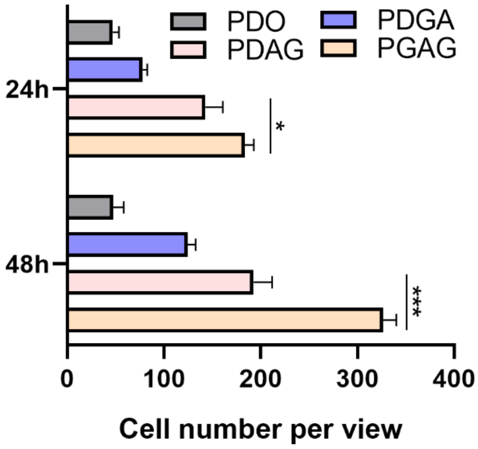

Figure S8. Cell number per view of HUVECs on PDO, PDGA, PDAG, PGAG membranes (n = 3). Data are presented as mean ± SD. p-values are calculated using one-way ANOVA with Bonferroni correction. *$p < 0.05$, **$p < 0.01$, and ***$p < 0.001$.

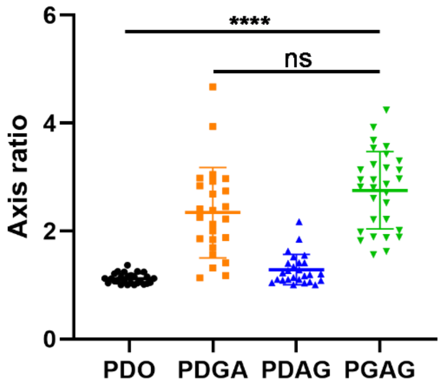

Figure S9. The axis ratio of HUVECs on PDO, PDGA, PDAG, PGAG membranes (n = 20). Data are presented as mean ± SD. p-values are calculated using one-way ANOVA with Bonferroni correction. ns=no significance, and ****p < 0.0001.

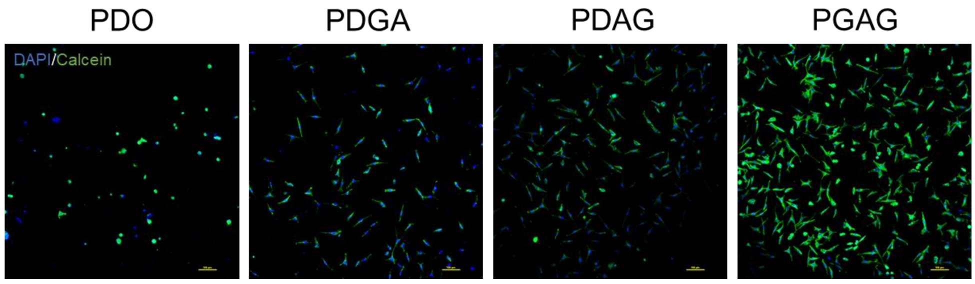

Figure S10. Immunofluorescence of L929 cells on PDO, PDGA, PDAG, PGAG membranes (blue: nuclear; green: phalloidin).

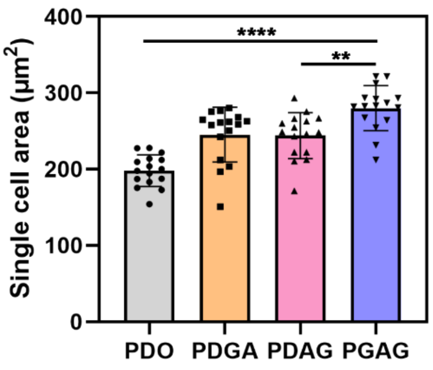

Figure S11. The single cell area of HUVECs on PDO, PDGA, PDAG, PGAG membranes (n =15). Data are presented as mean ± SD. p-values are calculated using one-way ANOVA with Bonferroni correction. *p < 0.05, **p < 0.01, ***p < 0.001, ****p<0.0001.

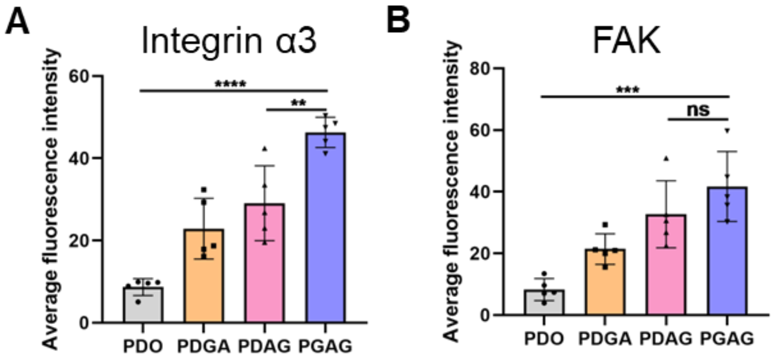

Figure S12. Quantification of average fluorescence intensity of integrin α3 (**A**) and FAK (**B**) (n = 5 for each test). Data are presented as mean ± SD. p-values are calculated using one-way ANOVA with Bonferroni correction. ns=no significance, *p < 0.05, **p < 0.01, ***p < 0.001, ****p<0.0001.

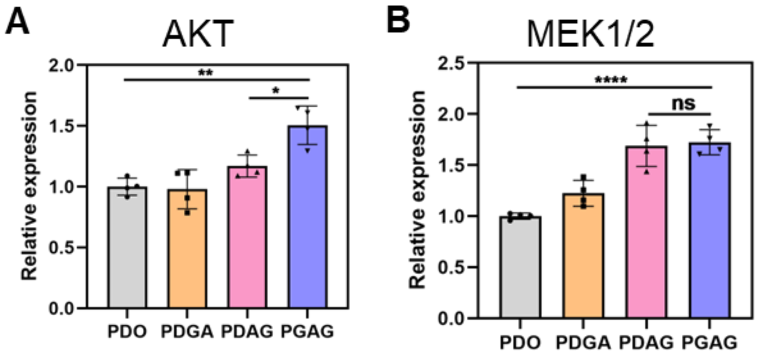

Figure S13. Statistical data of AKT (**A**) and MEK1/2 (**B**) determined by western blot (n = 4 for each test). Data are presented as mean ± SD. p-values are calculated using one-way

ANOVA with Bonferroni correction. ns=no significance, *p < 0.05, **p < 0.01, ***p < 0.001, ****p<0.0001.

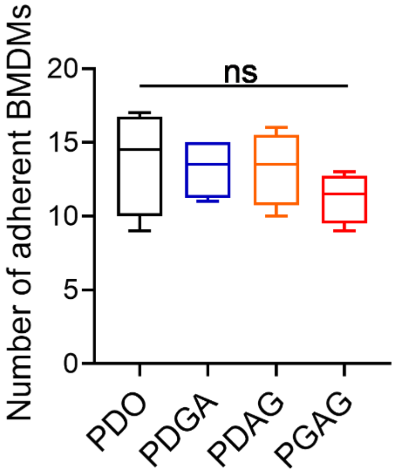

Figure S14. Number of adherent F4/80+ BMDMs on PDO, PDGA, PDAG and PGAG per view (n = 4). The box plot indicates the range from min to max. p-values are calculated using one-way ANOVA with Bonferroni correction. ns=no significance.

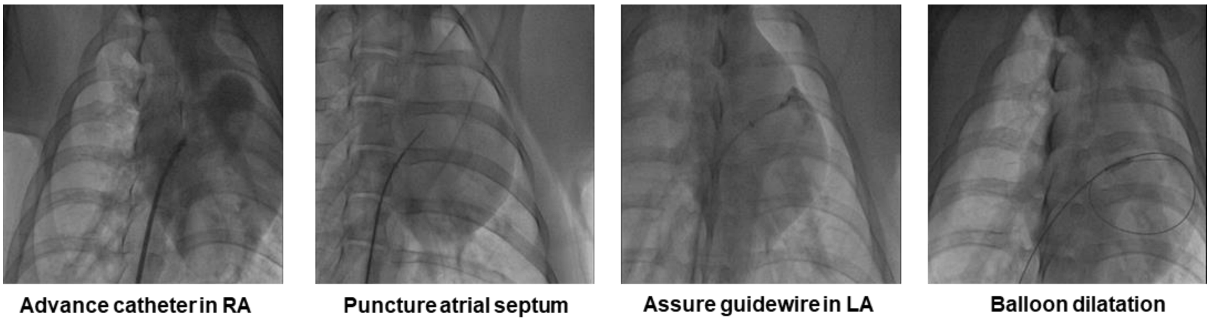

Figure S15. Procedure of procine ASD modelling.

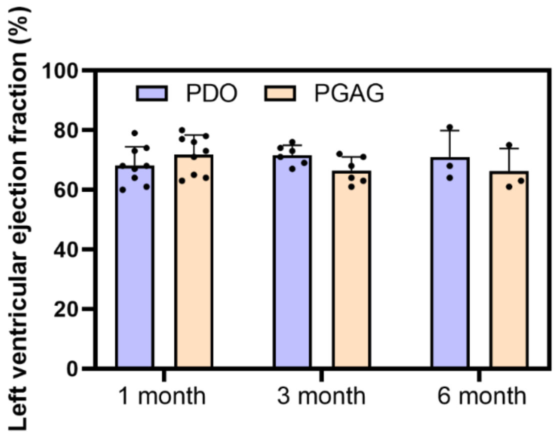

Figure S16. Left ventricluar ejection fraction of procine at 1, 3 and 6 months determined by echocardiography (n = 9 at 1 month, n = 6 at 3 month and n = 3 at 6 month). Data are presented as mean ± SD.

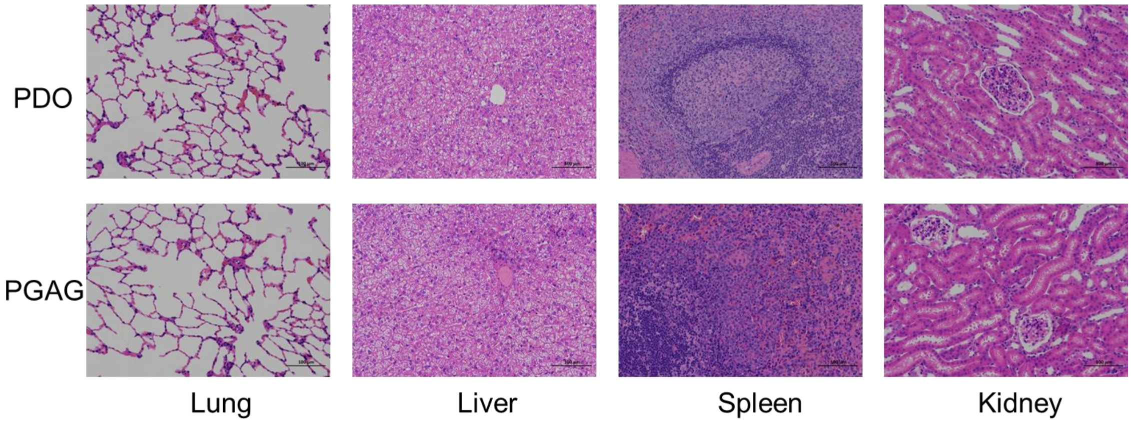

Figure S17. H&E staining of lung, liver, spleen, and kidney.

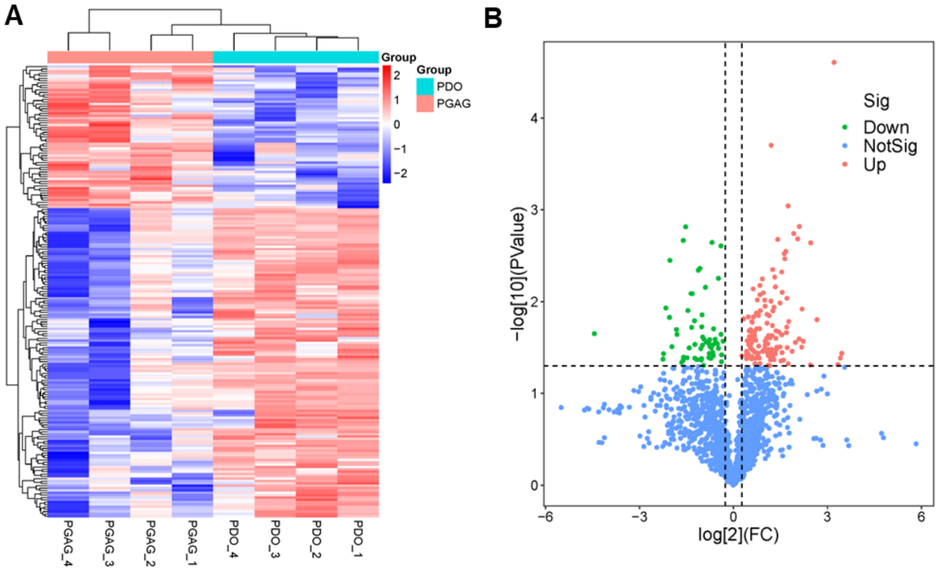

Figure S18. Proteomics of cardiac tissue surrounding occluders. (**A**) Cluster heatmap of significantly upregulated and down regulated DEPs. (**B**) Volcano plots. The red dots indicate the proteins selected based on adjusted P value < 0.05 and log2FC > 2.

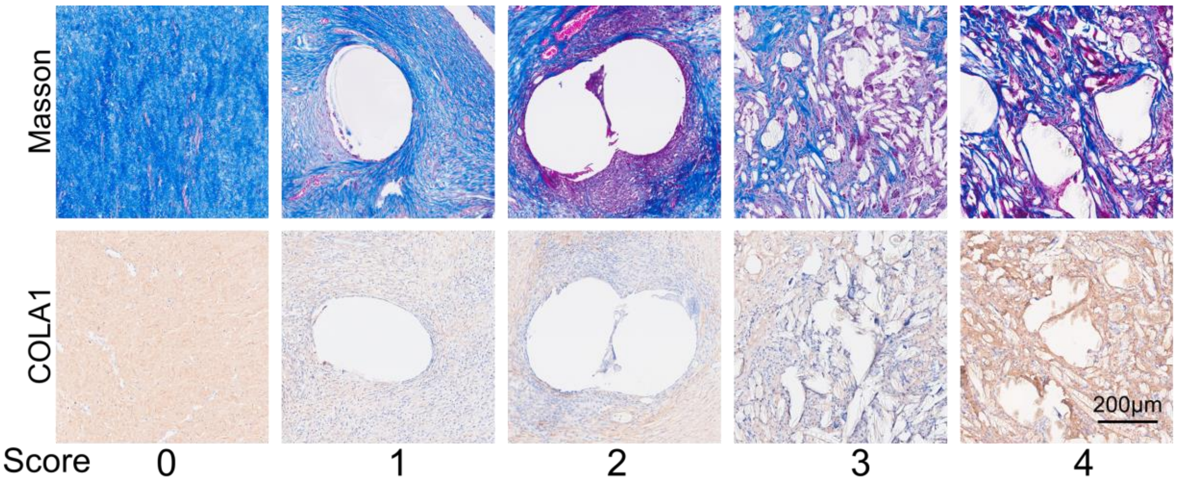

Figure S19. Fiber Disorder Score Evaluation Criteria. 0 = Order. Fibers exhibit a well-organized and compact structure, forming an integral tissue. 1 = Slight Disorder: Fibers consistently surround the materials with minimal interspace. The direction of fibers is harmonious. 2 = Mild Disorder: A mild chaos appears in the direction and arrangement of

surrounding fibers. 3 = Moderate Disorder: The direction becomes chaotic, and the interspace increases. 4 = Severe Disorder: The tissue displays the largest interspace with the most chaotic collagen deposition.
